# Supplementary material for: The causal role of circulating inflammatory markers in osteoporosis: a bidirectional Mendelian randomized study
Source: Front Immunol. 2024 Jul 18;15:1412298. doi: 10.3389/fimmu.2024.1412298 (PMC11291241; doi:10.3389/fimmu.2024.1412298)
Supplement: Supplementary file 1 [file DataSheet_1.zip › supplementary material/CIM-OSTEOPOROSIS /GCST90274823.csv_leave_one_out.pdf]

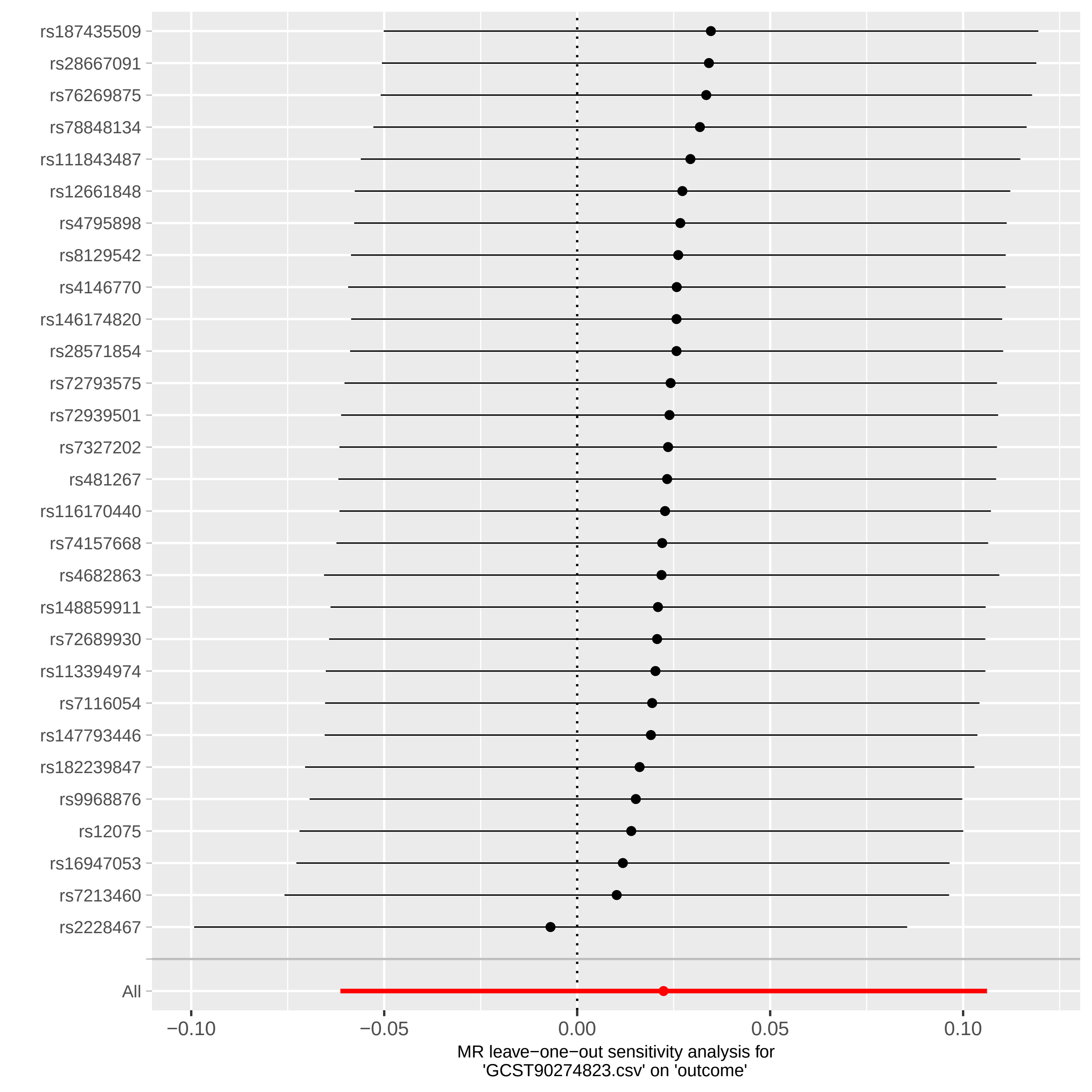
rs187435509
rs28667091
rs76269875
rs78848134
rs111843487
rs12661848
rs4795898
rs8129542
rs4146770
rs146174820
rs28571854
rs72793575
rs72939501
rs7327202
rs481267
rs116170440
rs74157668
rs4682863
rs148859911
rs72689930
rs113394974
rs7116054
rs147793446
rs182239847
rs9968876
rs12075
rs16947053
rs7213460
rs2228467
All
−0.10
−0.05
0.00
0.05
0.10
MR leave−one−out sensitivity analysis for
'GCST90274823.csv' on 'outcome'
